# Cultivation of *Staphylococcus epidermidis* in the Human Spaceflight Environment Leads to Alterations in the Frequency and Spectrum of Spontaneous Rifampicin-Resistance Mutations in the *rpoB* Gene

**DOI:** 10.3389/fmicb.2016.00999

**Published:** 2016-06-28

**Authors:** Patricia Fajardo-Cavazos, Wayne L. Nicholson

**Affiliations:** Department of Microbiology and Cell Science, University of Florida, Merritt IslandFL, USA

**Keywords:** antibiotic resistance, mutation, *rpoB*, rifampicin, *Staphylococcus epidermidis*, space flight

## Abstract

Bacteria of the genus *Staphylococcus* are persistent inhabitants of human spaceflight habitats and represent potential opportunistic pathogens. The effect of the human spaceflight environment on the growth and the frequency of mutations to antibiotic resistance in the model organism *Staphylococcus epidermidis* strain ATCC12228 was investigated. Six cultures of the test organism were cultivated in biological research in canisters–Petri dish fixation units for 122 h on orbit in the International Space Station (ISS) as part of the SpaceX-3 resupply mission. Asynchronous ground controls (GCs) consisted of identical sets of cultures cultivated for 122 h in the ISS Environmental Simulator at Kennedy Space Center. *S. epidermidis* exhibited significantly lower viable counts but significantly higher frequencies of mutation to rifampicin (Rif) resistance in space vs. GC cultures. The spectrum of mutations in the *rpoB* gene leading to Rif^R^ was altered in *S. epidermidis* isolates cultivated in the ISS compared to GCs. The results suggest that the human spaceflight environment induces unique physiologic stresses on growing bacterial cells leading to changes in mutagenic potential.

## Introduction

Planning is currently underway for future long-duration missions through interplanetary space to the Moon, near-Earth asteroids, or Mars (NASA, 2015). Both the National Research Council (NRC) and the International Space Exploration Coordination Group (ISECG) have assigned a high priority to studies aimed at better understanding astronaut health risks during space exploration; furthermore, these agencies have recognized the International Space Station (ISS) as the best available platform to conduct research activities to address these challenges (International Space Exploration Coordination Group (ISECG), 2013; National Research Council [NRC], 2014).

Studies conducted during short- and medium-duration missions (from a few days up to ~1 year) in vehicles such as Spacelab, Soyuz, Shuttle, Mir, and ISS have provided a wealth of information concerning astronaut health and function in the spaceflight environment of low-Earth orbit (LEO; Taylor, 2015). Based on the knowledge gained from these missions, NASA has developed successful risk mitigation strategies. However, long-duration missions (greater than 1 year) into interplanetary space are more likely to expose astronauts to new and higher risks to their health and performance than excursions to LEO, primarily due to: (i) chronic exposure to microgravity; (ii) increased exposure to ionizing radiation from solar and galactic sources; and (iii) extreme confinement and isolation (Reitz, 2008; NRC, 2011; Zeitlin et al., 2013; Taylor, 2015). One particular concern for planners of long-duration missions is the possibility of infectious disease (Scott-Conner et al., 2015; Taylor, 2015). Prolonged exposure to the stresses of spaceflight can lead to dysregulation of astronauts’ immune systems, thus increasing their susceptibility to infectious disease (Crucian et al., 2009; Guéguinou et al., 2009; Taylor, 2015). To minimize the possibility of bringing infectious disease agents onboard, pre-launch mitigation protocols are in place such as screening astronauts for certain microbes and irradiation of food (NASA, 2010). However, the essential nature of the human microbiome (Cho and Blaser, 2012) renders it impossible to remove all potentially infectious microorganisms from astronauts. Indeed, deterioration of astronauts’ immune systems in space has led to increased reactivation of latent viral infections by Varicella–Zoster, Epstein–Barr, and Cytomegalovirus during 12–16 day Shuttle flights (Mehta et al., 2014). Opportunistic bacterial infections of the urinary tract, upper respiratory tract, and subcutaneous tissue have been documented from Shuttle missions STS-1 to STS-108 (Sams, 2009), and conjunctivitis, respiratory tract, and dental infections were documented among long-term inhabitants of space station Mir (Ball and Evans, 2001).

Numerous studies have been conducted to understand how bacteria themselves respond to the human spaceflight environment, with particular regard to the possibility of their enhanced pathogenic potential or resistance to antimicrobial treatments [reviewed extensively in Rosenzweig et al. (2014)]. In some spaceflight experiments, it has been observed that spaceflight can lead to enhanced virulence in some bacteria (*Salmonella typhimurium, Escherichia coli*, and *Pseudomonas aeruginosa*), but not in others (*S. typhimurium, Staphylococcus aureus* MRSA, *Enterococcus faecalis*, and *Listeria monocytogenes*; note: in *S. typhimurium*, both cases have been observed in different spaceflight experiments; reviewed in Rosenzweig et al., 2014). Taken together, the data suggest that the combined phenomena of altered astronaut immunity and heightened virulence of some microbes in the spaceflight environment might lead to an increased incidence of astronaut infections by some opportunistic pathogens during extended missions (Klaus and Howard, 2006).

Space stations start out as basically clean environments, but upon habitation, they are rapidly colonized by numerous environmental and astronaut-associated species of microorganisms, which proceed to adapt and evolve in response to selective pressures unique to the spaceflight environment (Novikova, 2004; van Tongeren et al., 2007). A number of opportunistic pathogens have been isolated from space station crew quarters and from astronauts, including both Gram-positive genera (*Bacillus, Enterococcus, Staphylococcus*, and *Streptococcus*) and Gram-negative genera (*Citrobacter, Enterobacter, Escherichia, Flavobacterium, Haemophilus, Klebsiella, Morganella, Proteus, Pseudomonas, Ralstonia, Serratia, Stenotrophomonas*, and *Yersinia*; Ilyin, 2005; Klaus and Howard, 2006). From studies of astronauts on space stations Mir and ISS, it was documented that the microbial diversity of the upper respiratory and gastrointestinal tracts was lowered during spaceflight, while the proportion of opportunistic pathogens increased. In addition, extensive exchange of microbial flora of the upper respiratory and gastrointestinal tracts has been documented among astronauts sharing confined space habitats [reviewed in Taylor and Sommer (2005)]. Recent microbial monitoring of the ISS environment revealed that *Bacillus* and *Staphylococcus* spp., are the most ubiquitous organisms cultured from the ISS, being especially abundant in samples taken from crew quarters, vacuum debris, and HEPA filters (Venkateswaran et al., 2014; Checinska et al., 2015). Not surprisingly, *Staphylococcus epidermidis* was the most frequently encountered organism in the ISS microbiome, due to its close association with humans as a skin commensal (Venkateswaran et al., 2014).

As on Earth, antibiotics are the predominant treatment of choice for bacterial infections on human spaceflight missions. Knowledge about the efficacy of antimicrobial interventions to treat infections during spaceflight is limited, given the difficulty to assess the interplay between pharmacokinetics of antibiotics or the physiologies of host and microbiome, both of which are altered in the spaceflight environment (Wotring, 2012; Taylor, 2015). Decreased susceptibility of microbes to antibiotics has been observed in the space environment during experiments performed on *Salyut 7* (Tixador et al., 1985) and the Space Shuttles *Challenger* (Lapchine et al., 1986) and *Discovery* (Tixador et al., 1994).

Mutation is a common mechanism by which bacteria become resistant to antibiotics. A body of evidence indicates that the spectrum of spontaneous mutation to antibiotic resistance can be altered by the environment to which microbes are exposed (Nicholson and Maughan, 2002; Nicholson and Park, 2015), including the spaceflight environment (Yatagai et al., 2000). In the experiments described here, we chose to study mutations leading to resistance to the antibiotic rifampcin (Rif), because: (i) Rif is a clinically relevant antibiotic used singly and in combination to treat a wide variety of infections; and (ii) Rif^R^ mutations are easily identified by nucleotide sequencing, as they reside within small regions of the *rpoB* gene encoding the β-subunit of RNA polymerase (Severinov et al., 1993).

Taken together, the above observations indicate that the possibility must be considered of antibiotic-resistant strains emerging, and potentially becoming dominant types, in the microbiomes of crew members. Therefore, to explore the development of antibiotic resistance in a potential opportunistic pathogen during long-term human habitation in space, the present communication describes experiments using the biological research in canisters (BRIC) hardware in which growth of *S. epidermidis*, as well as the frequency and spectrum of mutation to Rif^R^, were measured after spaceflight on the ISS and compared to asynchronous ground controls (GCs).

## Materials and Methods

### Bacterial Strain, Media, and Growth Conditions

The strain used was *S. epidermidis* strain ATCC12228 obtained from the American Type Culture Collection, Manassas, VA, USA. Medium used throughout was trypticase soy yeast extract (TSY) medium consisting of (g/L): tryptone, 15; soytone, 5; NaCl, 5; yeast extract, 3; K_2_HPO_4_, 2.5; glucose, 2.5; and final pH 7. For semisolid plates, agar was added to TSY at 15.0 g/L. As appropriate, the antibiotic rifampicin (Rif; Sigma–Aldrich) was added to TSY at a final concentration of 5 μg/mL.

### Sample Preparation

*Staphylococcus epidermidis* cells were prepared by overnight growth in TSY liquid medium and a calibration curve was constructed relating Optical Density at 660 nm (OD_660_) to viable cell titer. Overnight cultures were diluted in TSY to a working concentration of 10^8^ CFU per mL prior to use. Aliquots of 0.1 mL (~10^7^ CFU) of the suspension were applied to the bottoms of sterile 60-mm diameter Petri dishes (Falcon cat. no. 1007) and air-dried for 48–72 h at room temperature prior to use.

### BRIC Spaceflight Hardware

The experiments described here utilized BRIC spaceflight hardware, which has been described in detail previously (Paul et al., 2012). BRIC canisters hold six 60-mm diameter Petri dish bottom halves in small subcompartments called Petri dish fixation units (PDFUs). Each PDFU allows for injection of medium, referred to as actuation, to initiate bacterial growth. For flight (FL) experiments, one BRIC canister was used containing six PDFUs. Immediately, adjacent to the FL canister was deployed a HOBO^®^ temperature data logger (Onset Computer Co., Bourne, MA, USA). Post-flight asynchronous GC experiments were conducted using the same hardware and configuration as in the FL experiment. Each PDFU was loaded with a Petri dish containing air-dried cells, and 13 mL of sterile TSY medium was loaded into a separate reservoir. To prevent contamination, all reagents and equipment used were sterilized prior to use and PDFUs were assembled using aseptic technique within a biological containment hood.

### Spaceflight Timeline

**FIGURE 1:**
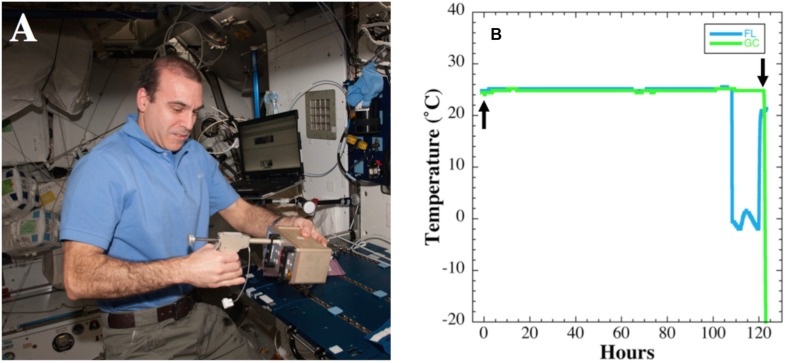
**(A)** Actuation of BRIC-18 canister by astronaut Rick Mastracchio aboard the ISS. **(B)** Temperature data from BRIC-18 flight (FL; blue line) and ground control (GC; green line) samples. Actuation time is denoted by the upward arrow and termination of experiment by moving to −80°C freezer is denoted by the downward arrow.

The BRIC–PDFU payload described above was the 18th BRIC mission to space, and was designated BRIC-18. The BRIC-18 hardware payload was launched on the third SpaceX cargo resupply mission to the ISS (SpaceX-3) on April 18, 2014, using the Falcon 9 rocket and Dragon capsule configuration. The Dragon capsule docked with the ISS on April 21, 2014. The BRIC canister was transferred to the ISS on April 22, 2014 and stowed in the US laboratory module. Actuation of the PDFUs was performed on April 30, 2014 at 10:08 EDT (**Figure 1A**) and samples were incubated at ISS ambient temperature (**Figure 1B**) until May 5, 2014 at 12:04 PM EDT, resulting in a total incubation time of 122 h. Growth was terminated by transfer of the BRIC canister to the onboard −80°C MELFI freezer. Samples were returned to Earth in the Dragon capsule on May 18, 2014 and were maintained in the frozen state until return to Kennedy Space Center (KSC) for de-integration and further processing.

### Ground Control Timeline

Asynchronous GC experiments were performed in a BRIC canister according to the timeline determined during the FL experiment. Samples were incubated in the KSC ISS Environmental Simulation Chamber following the temperature regime recorded during flight (**Figure 1B**), and growth was terminated by transfer to a −80°C freezer, where samples were stored until further processing.

### Post-Experiment Sample Processing

Both FL and GC BRIC canisters were transferred from storage at −80°C to a +4°C refrigerator and allowed to thaw overnight. Petri dishes were removed from PDFUs and cells were resuspended using sterile disposable rubber spatulas. Resuspended cultures were transferred to sterile 50-mL conical centrifuge tubes, and the total volume recovered was measured. For viable counts, aliquots from cultures were diluted serially 10-fold in TSY medium, dilutions plated on TSY, and colonies counted after incubation at 37°C for 24 h to obtain CFU/mL. To obtain the total CFU per PDFU, the CFU/mL were multiplied by the volume of liquid recovered. To select for Rif^R^ mutants, cultures were concentrated by centrifugation, plated without dilution onto TSY + Rif plates, and colonies counted after incubation at 37°C for 24 h. The frequency of mutation to Rif^R^ was calculated by dividing the total number of Rif^R^ mutants by the total number of viable cells from each culture. Individual Rif^R^ mutants were streak-purified on TSY + Rif plates and processed for DNA sequencing.

### DNA Sequencing and Analyses

Primers used for PCR amplification of two Rif^R^ regions of the *S. epidermidis rpoB* gene are listed in **Table 1.** The corresponding *rpoB* regions were amplified by PCR directly from cells as previously described (Nicholson and Maughan, 2002) and their nucleotide sequences determined at the University of Florida Interdisciplinary Center for Biotechnology Research (UF-ICBR). Multiple *rpoB* sequences were aligned using the online Clustal Omega server^1^ to identify the position of mutations relative to the wild-type *rpoB* sequence obtained in parallel from *S. epidermidis* ATCC12228.

**Table 1:** Oligonucleotide primers used for amplification of *rpoB* regions in *Staphylococcus epidermidis.*

Primer name	Sequence 5′ → 3′	*rpoB* region amplified
Sep rpoB – 13F	GTAAGGGTGAACACACAA	N-cluster
Sep rpoB + 685R	CTCTAATAAAGCCTGTTCTG	N-cluster
Sep rpoB + 1322F	CTATTACGCCACAACAACTC	Clusters I, II, III
Sep rpoB + 2023R	GCGTCCTCTATGCTTAGC	Clusters I, II, III

### Statistical Analyses

Non-parametric statistical parameters and tests of significance (Kruskal–Wallis) were computed on log_10_-transformed data using Kaleidagraph version 4.5.2 (Synergy Software, Reading, PA, USA).

## Results

### Temperature Data in FL vs. GC Experiments

Temperature data were logged at 10-min intervals during the FL and GC experiments and the data are presented in **Figure 1B.** The average temperature recorded during the growth period in FL was 25.1 ± 0.12°C until 107 h, at which time the immediately adjacent FL HOBO unit malfunctioned for the duration of the experiment (**Figure 1B**). However, the HOBO malfunction did not affect the course of the FL experiment, as other HOBO units in nearby BRIC canisters functioned correctly and confirmed a nominal temperature profile (data not shown). The average temperature in the GC experiment was 24.8 ± 0.16°C, differing from the FL experiment by only 0.3°C. The HOBO unit in the GC experiment performed nominally for the entire 122 h duration of the experiment (**Figure 1B**).

### *Staphylococcus epidermidis* Growth and Frequency of Mutation to Rif^R^ in FL vs. GC

**FIGURE 2:**
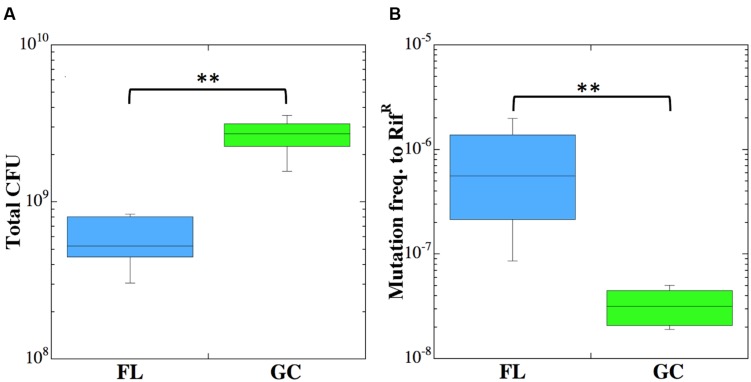
**Viable counts (CFU) (A) and frequency of mutation to Rif^R^ (B) of *Staphylococcus epidermidis* cultures incubated in FL (blue boxes) or GC (green boxes).** Double asterisks denote statistically significant differences (*n* = 6; Kruskal–Wallis; *P* < 0.01).

Viable counts of the cultures in all six PDFUs were determined for both FL and GC samples (**Figure 2A**). All *S. epidermidis* cultures grew to high titers in both FL and GC experiments, and viable counts were significantly higher in the GC cultures (**Figure 2A**). To determine the frequency of mutation to Rif^R^, cells from the FL and GC cultures were concentrated by centrifugation and plated onto TSY + Rif. FL cultures of *S. epidermidis* exhibited a 24-fold higher frequency of mutation to Rif^R^ than did GC cultures, and this difference was found to be highly significant (**Figure 2B**).

### Spectrum of Rif^R^ *rpoB* Mutations in FL vs. GC Experiments in *S. epidermidis*

**FIGURE 3:**
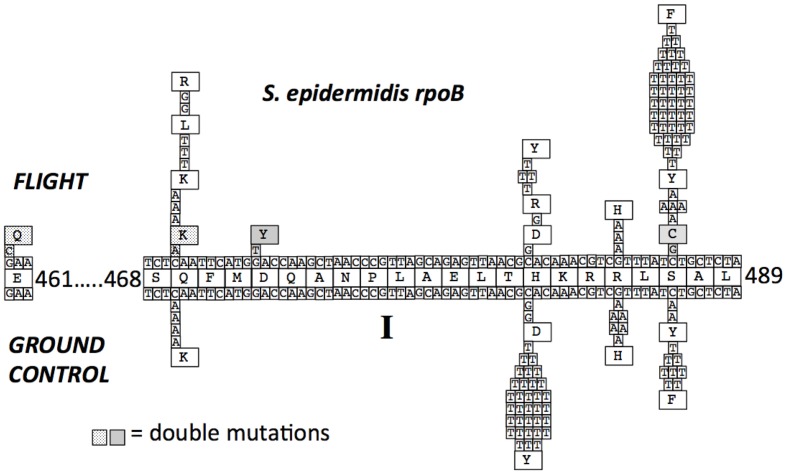
**Mutations in the *S. epidermidis rpoB* gene leading to Rif^R^.** The centerline depicts the wild-type RpoB amino acid sequence with coordinates. The lines above and below the amino acid sequence represent the wild-type DNA sequence of *rpoB*. Mutations found in Rif^R^ mutants obtained from FL and GC are denoted, as are the resulting deduced amino acid changes. Two double mutations found in the FL samples are indicated by stippled and shaded backgrounds.

Chromosomal DNA was isolated from a total of 67 FL and 57 GC Rif^R^ mutants, and the N-cluster and Clusters I, II, and III of their *rpoB* genes were amplified by PCR and sequenced. The data are presented in **Tables 2** and **3** and summarized graphically in **Figure 3.** Examination of the data revealed notable differences in the mutational spectrum within *rpoB* between the FL and GC samples.

**Table 2:** Summary of *rpoB* mutations leading to Rif^R^ in *S. epidermidis* flight (FL) vs. ground control (GC) samples.

PDFU	1		2		3		4		5		6		Total	
Mutation	FL	GC	FL	GC	FL	GC	FL	GC	FL	GC	FL	GC	FL	GC
Q469K		1							3	3			3	4
Q469L	1		1		1								3	0
Q469R			2										2	0
H482D										1	1	1	1	2
H482R			1										1	0
H482Y		2	1	7		8		8		4	3	4	4	33
R485H	2	3		1		1		1			1	1	3	6
S487F	12	1	4	1	7	1	10	1	6		3	4	42	8
S487Y	2			1	2					1	1		5	2
E460Q+Q469K			1										1	0
D472Y + S487C									1				1	0
No mutation found										1	1		1	1
Total sequenced	17	7	10	10	10	10	10	10	10	10	10	10	67	57

**Table 3:** Distribution of classes of Rif^R^ *rpoB* mutations appearing at least once in FL and GC samples^a^.

	FL						GC						No. of PDFUs	
PDFU number:	1	2	3	4	5	6	1	2	3	4	5	6	FL	GC
Amino acid change
Q469K					X		X				X		1	2
Q469L	X	X	X										3	0
Q469R		X											1	0
H482D						X					X	X	1	2
H482R		X											1	0
H482Y		X				X	X	X	X	X	X	X	2	6
R485H	X					X	X	X	X	X		X	2	5
S487F	X	X	X	X	X	X	X	X	X	X		X	6	5
S487Y	X		X			X		X			X		3	2
E460Q + Q469K		X											1	0
D472Y + S487C					X								1	0

*^a^Each class of mutation was scored as either present (X) or absent (blank) in the corresponding PDFU. Totals are tabulated in the rightmost two columns.*

#### *N*-Cluster

No mutations were detected in the N-cluster of the *S. epidermidis rpoB* gene in either the FL or GC samples.

#### Cluster I

All of the Rif^R^ mutations identified in both FL and GC samples of *S. epidermidis* were found to occur in Cluster I (**Table 2**; **Figure 3**). In agreement with previous reports (Severinov et al., 1993; Campbell et al., 2001; Nicholson and Maughan, 2002; Perkins et al., 2008), the most common amino acid changes in Cluster I leading to Rif^R^ were found at amino acids Q469, H482, and S487. At codon Q469, both FL (3/8) and GC (4/4) samples exhibited C-to-A transversions in the first position leading to a Q469K substitution (**Table 2**; **Figure 3**). However, FL samples also exhibited two changes at the second position of codon Q469; an A-to-T transversion and an A-to-G transition, leading to the amino acid substitutions Q469L and Q469R, respectively (**Table 2**; **Figure 3**). Together, these two mutations accounted for 5/8, or 63% of the total mutations found in FL samples, but were not represented at all in GC samples.

At codon H482 were found the majority of Rif^R^ mutations in GC samples (40/57 total, or 70%). In comparing the spectrum of Rif^R^ mutations between FL and GC samples, it was observed that all of the Rif^R^ mutations identified in the GC samples were found to occur at the first position of codon H482, either C-to-G transversions leading to substitution H482Y (33/35, or 94%), or C-to-T transitions leading to H482D (2/35, or 6%). FL samples also exhibited these same mutations (4/6, or 67% for H482Y; 1/6 or 17% for H482D, respectively; **Table 2**; **Figure 3**). In addition, a single mutation was identified at the second position of codon H482 only in FL samples; this was an A-to-G transition resulting in an H482R substitution (**Table 2**; **Figure 3**).

At codon R485, G-to-A transitions were detected at the second position in both FL and GC samples, leading to an R485H amino acid substitution at a slightly higher frequency in GC samples (7/57 total, or 12%) than in FL samples (3/67 total, or 4.5%; **Table 2**; **Figure 3**).

At codon, S487 was found the majority of Rif^R^ mutations in FL samples (47/67 total, or 70%). The largest proportion of Rif^R^ mutations in both FL samples (42/47, or 89%) and GC samples (8/10, or 80%) were found to consist of C-to-T transitions at the second position, resulting in the S487F substitution (**Table 2** and **Figure 3**). The remaining mutations at S487 consisted of C-to-T transitions at the second position, resulting in the amino acid substitution S487Y, found in 5/47 (11%) of FL samples and 2/10 (20%) of GC samples, respectively.

#### Double Mutations in FL Samples

Sequence analysis of Rif^R^ mutations in *S. epidermidis rpoB* revealed two double mutations in FL samples that were not detected in GC samples (**Table 2** and **Figure 3**). The first resulted in the double amino acid change D472Y + S487C and the second resulted in the double amino acid change E461Q + Q469K. While the single amino acid changes D472Y and Q469K have both been associated with Rif^R^ in other species, namely *E. coli* (Severinov et al., 1993) and *Bacillus subtilis* (Nicholson and Maughan, 2002), at present it is unclear if single mutations in *rpoB* leading to the E461Q or S487C substitutions by themselves can confer Rif^R^ in *S. epidermidis.*

#### Distribution of Rif^R^ *rpoB* Mutations by PDFU

Examination of **Table 2** indicated that in some PDFUs an unusually high number of repeats of the same Rif^R^ mutant were found. For example, 12 out of 17 Rif^R^ mutants sequenced from FL PDFU-1 were S487F, and 7 out of 10 mutants sequenced from GC PDFU-2 were H482Y (**Table 2**). These data suggested that some of the populations in both FL and GC samples contained “jackpots,” i.e., cultures in which the progeny of early-arising Rif^R^ mutants became over-represented in the final culture (Foster, 2006), which may have skewed the results reported in **Table 2** and their interpretation. We therefore re-analyzed the data by counting each type of mutation as either “present” (at least once) or “absent” in each PDFU, the results of which are presented in **Table 3.** When re-examined in this way, differences in the distribution of Rif^R^ mutants in FL vs. GC samples were still apparent. For example, the Q469L mutation was found in three FL PDFUs, but in zero GC PDFUs (**Table 3**). Conversely, present in nearly all GC PDFUs were the H482Y (6/6) and R485H (5/6) mutations but these mutations were present in only two FL PDFUs (**Table 3**). In addition, it appeared that the mutation leading to S487F was predominant in both FL (6/6) and GC (5/6) PDFUs, suggesting that mutation at this site was not affected by spaceflight (**Table 3**).

### Transition vs. Transversion Mutations in FL and GC Samples

As reported above, differences were observed in the location of *rpoB* mutations in FL vs. GC samples in *S. epidermidis* Rif^R^ mutants. These observations prompted us to examine the nature of nucleotide changes (i.e., transition vs. transversion mutations) in cells cultivated in spaceflight and on the ground. Examination of the distribution of transitions and transversions in *S. epidermidis* samples revealed the G:A and C:T transitions, and C:A and C:G transversions, were found both in FL and GC samples (**Table 3**). However, FL samples also exhibited A:G transitions and A:T, G:T, and G:C transversions that were not found in GC samples. Thus, the spectrum of mutational classes (transitions vs. transversions) was also altered in FL vs. GC samples.

## Discussion

The observations that (i) astronaut immune function becomes dysregulated during long-term spaceflight (Guéguinou et al., 2009; Crucian et al., 2014; Taylor, 2015); (ii) certain opportunistic bacteria can upregulate virulence functions in spaceflight (Rosenzweig et al., 2014; Taylor, 2015); and (iii) spaceflight can alter the antibiotic resistance profiles of bacteria (Lapchine et al., 1986; Tixador et al., 1994) have underscored the importance of studying the causes and consequences of the development of bacterial antibiotic resistance in the human spaceflight environment. Understanding how microgravity affects the development of not only bacterial antibiotic resistance, but of bacterial growth and metabolism in general, has been difficult to achieve despite substantial interest in the topic (reviewed in Nickerson et al., 2004; Klaus and Howard, 2006; Horneck et al., 2010). This results from the inherent limitations of performing microbiological research in spaceflight, due to infrequent opportunities to access spaceflight habitats, and limitations on the ability to perform large-scale, well-controlled, multireplicate, on-board experiments in the spaceflight environment. In particular, DNA damage, repair, and mutagenesis of bacteria inhabiting the human spaceflight environment remains largely unexplored. Previous studies of antibiotic resistance in microgravity have focused mainly on transient physiologic changes leading to increased or decreased antibiotic susceptibility (Tixador et al., 1985, 1994; Lapchine et al., 1986; Klaus and Howard, 2006). In contrast, the experiments reported here address the heritable emergence of antibiotic resistance in a microbe exposed to spaceflight stress resulting from mutations in genes encoding an important antibiotic target, RNA polymerase.

### Cell Numbers Achieved during Spaceflight

Experiments have been conducted in which the growth of various bacterial species, cultivated under otherwise-matching conditions of hardware, media, inoculation, etc., has been compared in spaceflight vs. GCs. A number of these studies have shown increased final cell density during spaceflight, while others have not observed significant differences between spaceflight and 1 x*g* conditions (reviewed in Benoit and Klaus, 2007; Horneck et al., 2010; Kim et al., 2013; Rosenzweig et al., 2014).

Attempts have been made to explain the phenomenon of bacterial growth to different densities in spaceflight vs. GCs. Benoit and Klaus (2007) suggested that microgravity abolishes convective mixing in liquid culture and that for some unknown reason non-motile cells grow better under these non-mixing conditions (Benoit and Klaus, 2007). According to their model, the movement of motile cells was proposed to mix the liquid medium, thus negating the lack of convective mixing in microgravity and leading to equal growth of motile cells in microgravity and 1 x*g* (Benoit and Klaus, 2007). However, Kim et al. (2013) recently explored the role of phosphate and/or oxygen availability, carbon source, and motility on the growth of *Pseudomonas aeruginosa* cells in spaceflight vs. GCs. They found that the carbon source used (citrate vs. glucose) did not result in significantly different growth in space vs. ground, but that cultivation under phosphate or oxygen limitation led to increased growth in spaceflight; furthermore, increasing phosphate concentration or aeration resulted in equal growth of cells in space vs. GCs (Kim et al., 2013). In addition, the effect of motility was tested by comparing the growth of the wild-type *P. aeruginosa* strain with a non-motile mutant harboring a deletion in the *motABCD* genes encoding the flagellar motor proteins. No significant difference in growth of the strains in space vs. ground was found, failing to support the “motility” hypothesis (Kim et al., 2013). The authors concluded that growth of *P. aeruginosa* to higher density in spaceflight was largely determined by oxygen and/or phosphate limitation, i.e., culture conditions (Kim et al., 2013).

In the experiments described here, we observed that *S. epidermidis* cells grew to significantly lower densities in FL samples than in GC samples. To the best of our knowledge, bacterial growth to a lower final concentration in spaceflight vs. GC samples has not been reported. The reason for this observation is not known, but our results also do not support the “motility” hypothesis, as *S. epidermidis* is a non-motile bacterium and grew to lower cell density in FL vs. GC samples, not higher as would be predicted. It is unlikely that availability of nutrients was limiting in these experiments, as the medium used (TSY containing 10% glycerol) is a rich medium. However, differences in diffusion of oxygen/CO_2_, nutrients, and/or potentially toxic end products in FL vs. GC cultures cannot be ruled out (see Possible Mechanisms).

### Frequency and Spectra of Mutations to Rif^R^ in FL vs. GC Samples

Few experiments have been performed to measure both the frequency and spectra of mutations in the same gene of microbes exposed to spaceflight in comparison to GCs, and none using *S. epidermidis*. In an earlier spaceflight experiment on space station Mir, it was noted that the frequency of mutation to streptomycin resistance (Str^R^) by *B. subtilis* spores was not significantly different between FL vs. GC samples, but that the spectrum of Str^R^ mutations in the *rpsL* gene was found to differ substantially (Yatagai et al., 2000). In the experiments reported here, we observed that *S. epidermidis* cells behaved somewhat differently, in that they both mutated to Rif^R^ with a significantly (24-fold) higher frequency in FL samples than in GC samples, and also displayed differences in their spectra of *rpoB* mutations. Although direct comparison between these two experiments (each using different organisms, growth conditions, and target genes) would be risky, the common finding of altered mutational spectra in both cases is intriguing and warrants further testing.

### Comparison of Simulated Microgravity vs. Spaceflight

Opportunities for true spaceflight experiments are very limited, and it is impossible on the Earth’s surface to generate a true microgravity environment. In response, investigators have turned to a number of ground-based systems that have been designed to simulate the effects of microgravity (reviewed in Anken, 2013). Among these, clinostats utilizing the rotating wall vessel (RWV) system have become widely used as spaceflight culture analogs (Nickerson et al., 2003; Anken, 2013). In some cases, alterations in bacterial gene expression and virulence have been found to correlate well between clinorotation and actual spaceflight experiments, but not in other cases (reviewed in Rosenzweig et al., 2014).

In a previous communication, we measured growth and mutation frequency to Rif^R^ in *S. epidermidis* cells grown in RWVs (Fajardo-Cavazos et al., 2014). In the RWV system, cultures of *S. epidermidis* grew to significantly higher titers in the vertical (i.e., simulated microgravity) orientation than in the horizontal (i.e., 1 x*g*) orientation (Fajardo-Cavazos et al., 2014). In stark contrast, in the present study, we found that *S. epidermidis* cells grew to significantly lower titers in space. We found further discordance when the frequency of mutation to Rif^R^ was compared between the spaceflight and RWV studies. In the present study, *S. epidermidis* FL cultures showed a highly significant increase in mutation to Rif^R^ than did GC samples, but in the RWV study, there was no statistical difference in mutation frequencies to Rif^R^ in vertical vs. horizontal RWVs (Fajardo-Cavazos et al., 2014). These results lead us to conclude that in the *S. epidermidis* model system described here, RWV experiments are of little value in predicting or modeling the behavior of this organism under spaceflight conditions.

### Possible Mechanisms

How does cultivation in spaceflight change the frequency and spectrum of *rpoB* mutations conferring Rif^R^ to *S. epidermidis*? In the experiments reported here, cells were exposed as nearly as possible to matched conditions of hardware, temperature, growth medium, incubation time, and times in stasis both before growth (as air-dried films) and after growth (frozen at –80°C). The two major factors prevailing in spaceflight are decreased gravity and increased ionizing radiation.

In orbit, objects inside or attached to the ISS are in free-fall such that the apparent gravitational force they experience is very small (around 1 μg). Microgravity affects a bacterial culture in a number of ways, for example: (i) buoyancy or sedimentation of cells is effectively negated; (ii) the rate of heat and mass transfer via passive convection becomes negligible, and in fact transfer of heat, nutrients, and waste toxins in a static culture becomes dominated by the rate of diffusion; and (iii) liquid behavior at solid surfaces and air–water interfaces is altered in microgravity, as surface tension, viscosity, capillary action, and wetting are removed from gravitational influence (Rosenzweig et al., 2014). The net result is that the same cells cultivated in matched hardware, medium, etc., in FL vs. GC could in fact be residing in two very different environments. Often, an increase in mutation rate can be a response to environmental stress (Galhardo et al., 2007), suggesting that cells in FL samples might be experiencing stress. One way of testing this hypothesis would be to measure global gene expression by performing whole-transcriptome, -proteome, and -metabolome analyses on FL vs. GC cultures, experiments which are currently in progress.

At the altitude of ISS orbit (~400 km), the interior of the vehicle is exposed to ionizing radiation consisting mostly of high-energy photons and atomic nuclei (Anonymous, 2002). The *S. epidermidis* FL samples inhabited the ISS interior for 30 days. Ongoing dosimetry indicates that the dose rate of ionizing radiation inside the ISS during this period amounted to ~13–14 mGy, compared to the global average background radiation dose on Earth of ~0.25 mGy during the same period (Puchalska et al., 2014). This dose is likely an overestimate for the FL samples, as they were also stored within lockers, inside aluminum BRIC canisters and aluminum-and-epoxy PDFUs. Nevertheless, FL samples were almost certainly exposed to a higher ionizing radiation dose than their GC counterparts. In support of the notion that FL samples may have received ionizing radiation hits, we observed two double mutations in *rpoB* from FL but not from GC samples; interestingly, induction of multiple closely linked mutations are a hallmark of ionizing radiation (Eccles et al., 2011).

### Broader Implications of the Results

In this communication, we report that cultivation of *S. epidermidis* in the human spaceflight environment of the ISS led to (i) an increase in the mutation frequency to Rif^R^ and (ii) alterations in the spectrum of mutations in the *rpoB* gene which result in Rif^R^. The results reported here with *S. epidermidis* appear to support previous results obtained using *B. subtilis* on space station Mir (Yatagai et al., 2000), and lead us to propose that exposure of bacteria to the human spaceflight environment can result in an alteration of the location of mutational hotspots within the target genes encoding antibiotic resistance factors. Further experiments using a greater variety of microorganisms will be needed to test this hypothesis.

The target gene used in this study, *rpoB*, encodes the β subunit of the enzyme RNA polymerase, a multisubunit enzyme which makes contacts with every transcribed gene in the bacterial genome. We and others have previously shown that single point mutations in *rpoB* leading to Rif^R^ can profoundly alter the global pattern of gene expression in bacteria and lead to (i) changes in growth, transformation, sporulation and spore resistance properties (Maughan et al., 2004; Moeller et al., 2012); (ii) activation of otherwise cryptic phenotypes (Perkins and Nicholson, 2008); and (iii) artificial triggering of the stringent response (reviewed in Alifano et al., 2015). It will be important to determine how the novel mutational changes in *rpoB* reported in this communication affect the physiological properties of the resulting mutants, such as their: growth and survival properties on environmental surfaces and in the host; communicability; pathogenic potential; and resistance to multiple antibiotics.

Management of microbial antibiotic resistance in confined environments on Earth, such as hospitals, prisons, military barracks, nursing homes, etc. has proven difficult. The results reported here suggest that our Earth-based understanding of the emergence of antibiotic resistance may not be wholly applicable to the human spaceflight environment. Research into the biology of spaceflight has uncovered alterations in crewmember immune function, microbial diversity, and in altered regulation of microbial physiologic responses, leading in some cases to increased virulence and antibiotic resistance (reviewed in Ott et al., 2012). In addition to the above factors, elucidation of the spaceflight-specific genetic mechanisms leading to the emergence of antibiotic resistance is needed to inform mitigation strategies for crew health and mission success. As more humans embark on longer sojourns into space, the more important these considerations will become.

## Author Contributions

PF-C and WN both contributed to the intellectual content, design and execution of the experiments, interpretation of the data, and writing of the manuscript, and are accountable for all aspects of the work described herein.

## Conflict of Interest Statement

The authors declare that the research was conducted in the absence of any commercial or financial relationships that could be construed as a potential conflict of interest.

## Footnotes

1 http://www.ebi.ac.uk/Tools/msa/clustalo/
